# Gamal Shiha: eliminating hepatitis C

**DOI:** 10.2471/BLT.24.030124

**Published:** 2024-01-01

## Abstract

Gamal Shiha talks to Gary Humphreys about how Egypt went from being one of the highest hepatitis C-prevalence countries in the world to being on the brink of eliminating the disease.

*Q: When did you encounter your first patients infected with hepatitis C?*

A: In the late 1980s, although at the time we referred to the cases as ‘non-A’ and ‘non-B’ because the hepatitis C virus (HCV) had not yet been identified. It was first identified in 1989 and the ELISA (Enzyme-Linked Immunosorbent Assay) test adapted to detect it, at which point it became clear that we had a real problem on our hands.

*Q: How big was the problem in epidemiological terms?*

A: In terms of prevalence, based on the positive HCV antibody tests that we were getting, we estimated that we were looking at 1 in 5 people infected or having been infected. We also had data from Egyptian workers returning from Saudi Arabia and other countries, 20% to 25% of whom tested positive for HCV antibodies. The first national prevalence surveys carried out in 1994 indicated a 14.5% prevalence rate among the general population. So, at that time, we had probably the highest prevalence of HCV in the world. Most of those infected were not even aware of the fact since, in the early stages of the disease, symptoms are generally minimal. It is only later that people may suffer from fatigue and fever, loss of appetite, nausea and abdominal pain. HCV-related deaths are primarily due to chronic or end-stage liver disease and liver cancer.

*Q: Why was prevalence so high?*

A: One of the key factors, as confirmed in a study published in *The Lancet* in March 2000, was a mass treatment campaign for schistosomiasis that peaked in the 1960s and 1970s. The government was pushing to improve the health of the population, especially in rural areas, and people were lining up in the villages to get injected with potassium antimonyl tartrate – the standard treatment for schistosomiasis at that time – with needles that were not properly sterilized. There were also many cases of oral infection from dental health services introduced as part of a government initiative to improve public health. Blood transfusion was another source of infection.

“There have been major developments on the treatment front.”

*Q: You set up the Association of Liver Patients’ Care (ALPC) in 1997, providing free medical services for those who could not afford treatment. What inspired that decision?*

A: A taxi driver. This was in 1995. I was treating people at the Mansoura University hospital, and this taxi driver came in with his wife, very sick. I diagnosed chronic hepatic encephalopathy (a brain and nervous system disorder) and told the man he had to stop driving because he was a danger to himself and others. I told him he needed medicine and a special diet. It was the reaction of his wife that struck me most. She said that without the taxi they would not be able to live, that I was basically killing them. That consultation made a profound impression on me, and I realized this was not just one person, this was hundreds of thousands of people. The next day I started talking to doctors and lawyers about setting up a non-governmental, non-profit organization to provide free hepatitis care. It took two years to bring it to fruition.

*Q: How did you go about setting it up and funding it?*

A: We started small. I found an apartment in a government building and got colleagues and local businessmen to contribute financially. The government officials I dealt with insisted on a separate entrance for the hepatitis clinic. So, I got my brother, who is an engineer, to put in another door. We offered free examinations and assays for patients who could not pay, and also free medication which at the time took the form of interferons and diabetes medication. People started to come in, and we put together symposia and lectures to raise awareness about how widespread the disease was. We ended up with six apartments in the same building. Today, we are in a bigger building and still going strong, meeting the needs of liver and digestive system patients with the latest international medical protocols and devices for diagnosing and treating diseases of the liver and digestive system. We currently do around 45 000 medical exams a year among other services, all for free to patients who are unable to pay. To provide a broader range of services, including surgical treatments, in 2011 we set up the Egyptian Liver Research Institute and Hospital (ELRIAH) as a subsidiary of the ALPC, in a purpose-built building. ELRIAH provides state of the art treatment and medical care, including surgical care, to around 50 000 hepatic patients per year.

*Q: Is ELRIAH also funded through donations?*

A: There is a mix of philanthropic funding and revenue derived from patients who can afford to pay, but we also have income from two big projects, the first of which is the institute for nursing and the second the institute for health, which offer training courses in laboratory analysis, radiology and medical equipment care. We are able to keep the ALPC and ELRIAH running with those different income streams.

*Q: ELRIAH was set up a year after the fall of President Hosni Mubarak’s government. To what extent did that event change the viral hepatitis landscape in Egypt?*

A: There has clearly been a change, but it is important to point out that Egypt, despite resource constraints, was strengthening its national preventive and treatment programmes prior to the revolution, notably with the establishment in 2006 of the National Committee for Control of Viral Hepatitis. But it’s true that there has been a significant focus on HCV with the administration put in place by President Abdel-Fattah El-Sisi in 2014. At the same time, there have been major developments on the treatment front with the approval of direct-acting antiviral (DAA) drugs such as sofosbuvir, which, in combination with other DAAs offers a 90%–95% cure rate using a well-tolerated 12-week oral regimen.

*Q: Sofosbuvir was first approved by the U.S. Food and Drug Administration in 2013 and retailed at 84 000 United states dollars (US$) per individual case. How were you able to afford it?*

A: We couldn’t afford it at that price, so I and my colleagues on the National Committee for Control of Viral Hepatitis negotiated with Gilead, the patent holder. That negotiation resulted in a voluntary licensing agreement in March 2014 under which sofosbuvir was sold to the Egyptian government at a steep discount, resulting in a cost of US$ 900 per treatment course.

*Q: How did you get them to bring the price down so dramatically?*

A: I told them that our ceiling for the entire treatment course was US$ 1000. I asked them to give us the same discount that they had given Nelson Mandela for antiretrovirals in South Africa. I don’t why they did it, but they did. Sofosbuvir was subsequently licensed to several Egyptian pharmaceutical companies, giving them the right to manufacture it domestically using active ingredients imported from China and India. Competition between producers has since brought the price down to around US$ 84 for the typical 12-week course.

“We have the tools to defeat this disease.”

*Q: The focus of your institutional work has been on the ALPC and ELRIAH, both of which are located near Mansoura, two hours north of Cairo. How have you been able to reach communities in rural areas?*

A: In collaboration with colleagues, I launched the “Towards a village free from viral hepatitis” campaign in 2015, using an educate, test and treat model. Between June 2015 and April 2019 we rolled the campaign out in 100 villages across the country. More than 250 000 villagers were screened for HCV and hepatitis B (HBV), and a total of 16 500 infected patients were given free treatment. Patients also received health education to promote safe behaviours and practices in the community to further reduce transmission and new infections. I was also asked to lead the education and scientific committee in parliament in 2016, and the development of the “100 million healthy lives” initiative which ran from October 2018 to April 2019.

*Q: Why 100 million lives?*

A: It was approximately the population of Egypt at that time, and was essentially a commitment to health for all. The aim was to offer free voluntary HCV screening to all residents aged 18 years and older, and to provide free treatment for people with confirmed infections. It was a truly massive undertaking with more than 60 000 health care personnel, including doctors, nurses, pharmacists, lab technicians and data entry employees, working at over 5 000 HCV screening and treatment sites.

*Q: How many people did you manage to screen and treat?*

A: In the end, nearly 50 million Egyptians and 36 000 foreign residents were screened. Of those, 2.2 million individuals were diagnosed seropositive, indicating prior HCV exposure or chronic infection, and referred for confirmatory testing. Of those referrals, 1.6 million patients were confirmed to have chronic HCV infection. Many of them were unaware of their status for the reasons I already mentioned. Some 900 000 patients were treated during the campaign and another 700 000 after its conclusion. Since then, we have tested over 60 million people and treated more than 4 million, reducing the incidence of new infections from 300 per 100 000 in 2014 to 9 per 100 000 in 2022. This is very close to the 2030 target set under the *Global health sector strategy on HIV, viral hepatitis and sexually transmitted infections* of less than 5 per 100 000 new cases per year.

*Q: Only 11 countries are on course to meet their goals for the elimination of hepatitis C by 2030. What can others learn from Egypt’s experience?*

A: The key barriers to achieving HCV global elimination targets have been identified as finding patients without symptoms, linking them to care, and providing access to affordable treatments. In Egypt we have shown that each of these obstacles can be overcome with political support, sustainable funding, community outreach and education, and ensuring access to diagnosis and treatment. Egypt is committed to working alongside our international brothers and sisters through collaborations such as the World Hepatitis Alliance, which is working on advocacy and awareness-raising and represents 249 members from 84 countries worldwide. We have the tools to defeat this disease, all we need is the will to do it.

